# Anti-CD6 monoclonal antibody UMCD6 induces CD6 internalization, remodels costimulatory receptor availability, and modulates CD4^+^ T-cell activation

**DOI:** 10.3389/fimmu.2026.1918840

**Published:** 2026-08-19

**Authors:** Kohei Maeda, Phillip L. Campbell, Qi Wu, Pei-Suen Tsou, Laura A. Cooney, Yang Mao-Draayer, David A. Fox, Mikel Gurrea-Rubio

**Affiliations:** 1 Division of Rheumatology, Department of Internal Medicine, University of Michigan, Ann Arbor, MI, United States; 2 Division of Pathogenesis and Translational Medicine, Department of Clinical Pharmacy, Showa Medical University Graduate School of Pharmacy, Tokyo, Japan; 3 Multiple Sclerosis Center of Excellence, Oklahoma Medical Research Foundation, Oklahoma City, OK, United States; 4 Autoimmunity Center of Excellence, University of Michigan, Ann Arbor, MI, United States

**Keywords:** CD2, CD28, CD4+ T-cell activation, CD6, UMCD6

## Abstract

**Introduction:**

CD6 is an immunological synapse-associated receptor that integrates adhesive and signaling cues to shape T-cell activation. We previously showed that, in CD6-humanized mice, the anti-human CD6 monoclonal antibody UMCD6 suppresses disease severity and Th1/Th17 responses in models of autoimmunity, but its mechanisms of action in human T cells remained incompletely defined.

**Methods:**

Primary human peripheral blood mononuclear cells and isolated T cells were used. UMCD6-induced changes in the surface expression, internalization, and localization of CD6, CD2, and CD28 on CD4+ T cells were analyzed by conventional and imaging flow cytometry. Functional effects of UMCD6 were assessed by measuring activation markers, intracellular IL-2 production, Ki-67 expression, and cytokine secretion following anti-CD3/CD28 stimulation. Proximal T-cell receptor signaling was evaluated by LAT phosphorylation and Indo-1–based calcium flux analysis, whereas PKC-θ phosphorylation was assessed by phospho-flow cytometry as a readout of downstream costimulatory signaling.

**Results:**

UMCD6 induced remodeling of the T-cell surface receptor landscape, including reduced surface CD6 and altered surface expression of CD2 and CD28. Imaging flow cytometry demonstrated redistribution and internalization of CD6 and CD2, accompanied by more modest changes in CD28 localization. Limited colocalization of CD6 with either CD2 or CD28 suggested broader reorganization of the costimulatory receptor network rather than passive co-internalization. Functionally, UMCD6 suppressed activation-induced expression of CD25, CD69, and OX40, reduced intracellular IL-2 production, and decreased secretion of IL-2, TNF-α, IFN-γ, and IL-17A, with more pronounced effects in CD4+ than CD8+ T cells. UMCD6 preserved proximal T-cell receptor signaling, including LAT phosphorylation and calcium flux, but was associated with a modest reduction in the early peak fold induction of PKC-θ phosphorylation.

**Discussion:**

These findings support a model in which UMCD6 remodels the availability and organization of costimulatory receptors rather than inducing simple co-internalization of CD6 with CD2 or CD28. This receptor reorganization, together with a modest reduction in PKC-θ-associated signal amplification, may contribute to the preferential suppression of CD4+ T-cell activation without broadly disrupting proximal T-cell receptor signaling.

## 1 Introduction

CD6 is a 105–130 kDa type I transmembrane glycoprotein of the scavenger receptor cysteine-rich (SRCR) superfamily that is constitutively expressed on mature T cells, a subset of natural killer (NK) cells, and select B-cell populations (1, 2). Beyond its original characterization as a lymphocyte surface marker, CD6 is now recognized as a multifunctional immunoregulatory receptor that can regulate T-cell activation, differentiation, and effector function through both adhesive and signaling-dependent mechanisms (3). Structurally, the extracellular region of CD6 contains three SRCR domains that mediate ligand binding, whereas the long cytoplasmic tail lacks intrinsic enzymatic activity but serves as a signaling scaffold through phosphorylation-dependent recruitment of adaptor proteins (4, 5). Accordingly, CD6 has emerged as a key regulator of immune synapse organization and T-cell receptor (TCR)-driven responses, with context-dependent effects on lymphocyte activation and immune homeostasis.

The best-characterized ligand of CD6 is activated leukocyte cell adhesion molecule (ALCAM/CD166), which binds to membrane-proximal domain 3 of CD6 (6, 7). Engagement of CD6 by ALCAM on antigen-presenting cells (APCs) promotes stable T cell-APC conjugate formation and contributes to the assembly and maintenance of the immunological synapse (8–10). Accumulating evidence from human disease and experimental models indicates that CD6 plays an important role in the pathogenesis of immune-mediated inflammatory disorders, including multiple sclerosis (11), rheumatoid arthritis (12), psoriasis (13), and other chronic inflammatory conditions (2). Mechanistically, CD6 has been linked to the regulation of T-cell activation thresholds (14), costimulatory signaling (15), and helper T-cell polarization, particularly in pathogenic Th1 and Th17 cells (16, 17). In line with these observations, our previous studies demonstrated that CD6 contributes to disease pathogenesis in murine models of multiple sclerosis (18), rheumatoid arthritis (19), and uveitis (20). In these systems, both genetic deletion of CD6 and treatment of CD6-humanized mice with UMCD6, a mouse anti-human monoclonal antibody directed against membrane-distal domain 1 of CD6, resulted in marked clinical improvement accompanied by significant suppression of Th1/Th17 responses. These findings support the concept that CD6 is not only a structural component of the immunological synapse, but also a functionally important regulator of differentiation pathways of effector T-cells involved in pathogenic CD4^+^ T-cell responses.

In parallel with its role in CD4^+^ T-cell-driven inflammation, we recently showed that CD6 can also support antitumor immunity mediated by cytotoxic lymphocyte (21, 22). Specifically, we identified CD318 as a novel ligand of CD6 expressed by multiple human tumor types, including breast, lung, and prostate cancer cells (23). In co-culture assays, the monoclonal antibody UMCD6 significantly enhanced lymphocyte-mediated tumor cell killing. In *SCID/beige* immunodeficient mice bearing human breast and prostate cancer xenografts, UMCD6 treatment suppressed tumor growth and prolonged survival. Mechanistically, these antitumor effects were associated, at least in part, with direct engagement of CD6 on CD8^+^ T cells and NK cells, leading to increased expression of the activating receptor NKG2D, decreased expression of the inhibitory receptor NKG2A, and enhanced induction of cytotoxic effector molecules such as perforin and granzyme B. Thus, in contrast to its inhibitory effects on pathogenic CD4^+^ T-cell responses, targeting CD6 with UMCD6 is known to augment cytotoxic lymphocyte function towards a wide range of cancer cells.

Together, these observations support a model in which CD6 serves as a lineage- and context-dependent immune rheostat, capable of exerting distinct effects in helper versus cytotoxic lymphocyte subsets. However, while the immunostimulatory consequences of UMCD6 on CD8^+^ T cells and NK cells have begun to be defined, the molecular basis by which UMCD6 suppresses CD4^+^ T-cell activation remains incompletely understood. In particular, it is unclear how engagement of membrane-distal domain 1 of CD6 influences proximal TCR and CD28 signaling, membrane organization of key costimulatory receptors, and downstream activation programs in conventional CD4^+^ T cells.

Based on these observations, we hypothesized that UMCD6 suppresses CD4^+^ T-cell responses by remodeling critical signaling pathways that govern T-cell activation and costimulation. In the present study, we sought to define the molecular mechanisms underlying the inhibitory effects of UMCD6 on CD4^+^ T cells by determining whether CD6 targeting alters the surface expression and spatial distribution of costimulatory molecules, modulates activation marker expression and cytokine production, and differentially affects TCR- and costimulatory signaling pathways. Using imaging flow cytometry, phospho-flow cytometry, and calcium flux analyses, we aimed to delineate how UMCD6 reshapes intracellular signaling networks in CD4^+^ T cells and to establish the mechanistic basis for its dual immunomodulatory activity.

## 2 Materials and methods

### 2.1 Antibodies

UMCD6 is a mouse anti-human CD6 monoclonal antibody generated in our laboratory that recognizes membrane-distal SRCR domain 1. UMCD6 was affinity-purified from mouse ascites using NAb™ Protein G Spin Columns (Thermo Scientific, Cat# 89953) and subsequently desalted by column chromatography using Pierce™ Dextran Desalting Columns (Thermo Scientific, Cat# PI43233) according to the manufacturer’s instructions. UMCD6 is a mouse IgG1κ monoclonal antibody, and a laboratory-produced mouse IgG1κ isotype- and concentration-matched antibody was used consistently as the control in all experiments. Commercially purchased antibodies are listed in **Table 1**. For stimulation of primary T cells, plate-bound anti-CD3 antibody was prepared by adding anti-CD3 antibody diluted to 5 µg/mL in PBS to the wells of a tissue culture plate and incubating overnight at 4 °C.

**Table 1:** List of antibodies.

Target	Clone	Fluorochrome	Vendor	Cat#	Application
Anti-rabbit IgG (H+L), F(ab’)2 Fragment	n/a	Alexa Fluor 488	Cell Signaling Technology	4412S	Flow cytometry
CD134	Ber-ACT35	Brilliant Violet 605	BioLegend	350027	Flow cytometry
CD14	MφP9	APC/Cyanine7	BD Pharmingen	557831	Flow cytometry
CD16	3G8	APC/Cyanine7	BioLegend	302017	Flow cytometry
CD19	HIB19	APC/Cyanine7	BioLegend	302218	Flow cytometry
CD2	TS1/8	FITC	BioLegend	309205	Flow cytometry
CD2	S5.2	BUV496	BD OptiBuild	750642	Flow cytometry
CD25	BC96	Alexa Fluor 647	BioLegend	302617	Flow cytometry
CD28	CD28.2	APC	BioLegend	302912	Flow cytometry
CD28	CD28.2	PE/Cyanine7	BioLegend	302926	Flow cytometry
CD3	HIT3a	Alexa Fluor 700	BioLegend	300324	Flow cytometry
CD3	UCHT1	RB613	BD Horizon	571085	Flow cytometry
CD3	UCHT1	Pacific Blue	BioLegend	300431	Flow cytometry
CD4	RPA-T4	PE-eFluor 610	Invitrogen	61-0049-42	Flow cytometry
CD4	OKT4	PerCP/Cyanine5.5	BioLegend	317428	Flow cytometry
CD4	OKT4	Brilliant Violet 510	BioLegend	317444	Flow cytometry
CD4	SK3	PE/Cyanine7	BioLegend	344611	Flow cytometry
CD56	5.1H11	APC/Cyanine7	BioLegend	362512	Flow cytometry
CD6	BL-CD6	PE	BioLegend	313906	Flow cytometry
CD69	FN50	BUV563	BD OptiBuild	748764	Flow cytometry
CD8a	RPA-T8	APC/Cyanine7	BioLegend	301016	Flow cytometry
CD8a	SK1	Pacific Blue	BioLegend	344717	Flow cytometry
CD8a	HIT8a	APC	BioLegend	300912	Flow cytometry
CD8a	RPA-T8	RB780	BD Horizon	568684	Flow cytometry
CD8a	SK1	PerCP/Cyanine5.5	BioLegend	344710	Flow cytometry
Granzyme B	GB11	Pacific Blue	BioLegend	515408	Flow cytometry
IL-2	W19046A	APC	BioLegend	310605	Flow cytometry
Ki-67	20Raj1	BUV737	Invitrogen	367-5699-42	Flow cytometry
Phospho Akt (Ser473)	A21001C	PE	BioLegend	606553	Flow cytometry
Phospho c-Fos (Ser32)	A22009C	Alexa Fluor 647	BioLegend	631257	Flow cytometry
Phospho c-Jun (Ser73)	A19012A/R/C	Alexa Fluor 488	BioLegend	605553	Flow cytometry
Phospho LAT (Tyr171)	A20005D	Alexa Fluor 647	BioLegend	946603	Flow cytometry
Phospho mTOR (Ser2448)	O21-404	Alexa Fluor 647	BD Phosflow	564242	Flow cytometry
Phospho NF-kB (p65) (Ser529)	A21012B	PE	BioLegend	614153	Flow cytometry
Phospho PI3K p85/p55 (Tyr458, Tyr199)	PI3KY458-1A11	APC	Invitrogen	MA5-36956	Flow cytometry
Phospho-PKCθ (Thr538)	Polyclonal	Unconjugated	Cell Signaling Technology	9377	Flow cytometry
CD28	CD28.2	Unconjugated	BioLegend	302934	T cell activation
CD3	OKT3	Unconjugated	BioLegend	317326	T cell activation
Anti-mouse IgG	n/a	HRP	Cell Signaling Technology	7076	Western blot
Anti-rabbit IgG	n/a	HRP	Cell Signaling Technology	7074	Western blot
GAPDH	14C10	Unconjugated	Cell Signaling Technology	2118	Western blot
Histone H3	Polyclonal	Unconjugated	Cell Signaling Technology	9715	Western blot
NFATC1	7A6	Unconjugated	Invitrogen	MA3-024	Western blot

### 2.2 Isolation and culture of PBMCs and T cells

Blood samples were collected from healthy volunteers at the University of Michigan. PBMCs were isolated by density-gradient centrifugation using Ficoll-Paque PLUS (Cytiva, Cat# 17144002). CD4^+^ T cells were negatively isolated from PBMCs using EasySep™ Human CD4^+^ T Cell Isolation Kit (STEMCELL Technologies, Cat# 17952). Cells were cultured in RPMI 1640 media (Corning, Cat# 10-040-CV) supplemented with 10% FBS, and 1x Antibiotic-Antimycotic (Gibco, Cat# 15240062) in a 37 °C and 5% CO2 incubator.

### 2.3 Flow cytometry

Cultured cells were washed with cell staining buffer (BioLegend, Cat# 420201), and treated with FcR blocker (Human TruStain FcX, BioLegend, Cat# 422302) and fixable viability dye (FVD) (Zombie Yellow™ Fixable Viability Kit, BioLegend, Cat# 423103) for 15 minutes prior to cell staining. These cells were then stained with appropriate antibodies for 30 minutes. After staining, the cells were fixed using BD Cytofix Buffer (BD Biosciences, Cat# 554655) and left at 4 °C until analysis.

For intracellular staining, the cultured cells were treated with brefeldin A (BioLegend, Cat# 420601) (final concentration, 5 μg/mL) for 4 hours prior to cell harvest. These cells were fixed and permeabilized with the Foxp3/Transcription Factor Staining Buffer Set (Invitrogen) according to the manufacturer’s protocol. For simultaneously analyzing the surface antigens and intracellular cytokines, the surface antigens were prestained for 30 min prior to performing the fixation/permeabilization process with appropriate fluorochrome-labeled antibodies.

Unstained cells and/or fluorescence minus one (FMO) controls were prepared to accurately set gates for positive populations. Dead cells and cell debris were excluded via forward versus side scatter gating and gating out of FVD-bright populations. Stained cells were analyzed using the Cytek Aurora or Cytek Aurora Evo (Cytek Biosciences). FlowJo software (v10.10.0) was used for data analysis and generating scatter plots and histograms. Gating strategies for each experiment are shown in **Supplementary Figure 1**.

### 2.4 Imaging flow cytometry

PBMCs from healthy donors were treated with UMCD6 or IgG control antibody (10 μg/mL) for indicated time periods. Cells were then stained for surface CD4 and CD8 using anti-CD4-PE-eFluor 610 and anti-CD8-Pacific Blue antibodies, followed by fixation and permeabilization with the Foxp3/Transcription Factor Staining Buffer Set (eBioscience). Subsequently, CD2, CD6, and CD28 were stained using anti-CD2-FITC, anti-CD6-PE, and anti-CD28-APC. Samples were analyzed on an ImageStreamX Mk II imaging flow cytometer (Cytek Biosciences), and cell images were acquired. Data analysis was performed using IDEAS software (Cytek Biosciences).

For internalization analysis, focused single-cell events were first selected, followed by gating on the indicated T-cell subsets. CD2^+^, CD6^+^, or CD28^+^ single-positive cells were then gated and plotted using Delta Centroid XY and Mean Pixel features. The gating strategy was generated using the IDEAS Feature Finder tool to identify image-based parameters that distinguished internalized from non-internalized fluorophore-staining patterns. Gating thresholds were then defined by visual confirmation of fluorophore localization in representative image galleries. Cells classified as internalized showed predominantly intracellular/perinuclear fluorophore localization, whereas less-internalized cells showed predominantly membrane-associated staining. Once established, the same gating thresholds were applied across treatment conditions and donors. For sample batches acquired on different days, the internalization gates were verified using representative image galleries to confirm consistent classification of internalized and non-internalized staining patterns.

For colocalization analysis of CD2 and CD6 or CD28 and CD6, double-positive cells, CD2^+^CD6^+^ or CD28^+^CD6^+^, were pre-gated. Colocalization was quantified using the Bright Detail Similarity feature in IDEAS software. Cells with a Bright Detail Similarity score ≥ 2 were defined as colocalized.

### 2.5 Quantitative real-time PCR

Total RNA was extracted from cultured cells using TRIzol Reagent (Invitrogen, Cat# 15596018) and the Direct-zol RNA Miniprep Kit (Zymo Research, Cat# R2052), according to the manufacturer’s instructions. RNA purity and concentration were assessed using a NanoDrop 2000 spectrophotometer (Thermo Fisher Scientific). Complementary DNA was synthesized from 500 ng of total RNA using the Verso cDNA Synthesis Kit (Thermo Fisher Scientific, Cat# AB1453B) following the manufacturer’s protocol. qRT-PCR was performed on a QuantStudio 5 Real-Time PCR System (Applied Biosystems) using Power SYBR Green PCR Master Mix (Applied Biosystems, Cat# 4367659). Primer sets for *CD2* and *CD28* were purchased from Sino Biological (Cat# HP100947 and HP101394, respectively). Additional gene-specific primers were synthesized by Integrated DNA Technologies. The primer sequences for *CD6* were as follows: forward, 5′-CCCATCACCATCCCCAAAG-3′; reverse, 5′-TGCAACTCTTCAAGTCCTTCC-3′. The primer sequences for *IL2* and *IL2RA* were based on previous reports (24, 25) and were as follows: *IL2RA*, forward 5′-GAGAAAGACCTCCGCTTCAC-3′ and reverse 5′-CGAGTGGCTAGAGTTTCCTG-3′; *IL2*, forward 5′-TACAGGATGCAACTCCTGTCTTGCATT-3′ and reverse 5′-GTTGCTGTCTCATCAGCATATTCACAC-3′. Samples were run in duplicate, and relative gene expression levels were calculated using the 2^-ΔΔCt^ method, normalized to β-actin. Results are presented as fold changes relative to IgG-pretreated cells.

### 2.6 Activation-induced marker assay

PBMCs pretreated with UMCD6 or an isotype-matched IgG control antibody (10 μg/mL) for 24 hours were activated with either plate-bound anti-CD3 antibody alone or a combination of plate-bound anti-CD3 and soluble anti-CD28 antibody (5 µg/mL). Following activation, both cells and culture supernatants were collected. Cells were analyzed by flow cytometry to assess the expression of activation-induced markers, and supernatants were used for ELISA to quantify cytokine levels using the DuoSet ELISA Kit for human IL-2 (Cat# DY202), TNF-alpha (Cat# DY210-05), IFN-gamma (Cat# DY285B-05), IL-4 (Cat# DY204-05), and IL-17 (Cat# DY317-05) (R&D Systems). To define gates for marker-positive populations, unstimulated cells were used as a negative control, whereas cells stimulated with phorbol 12-myristate 13-acetate (PMA) (200 nM) and ionomycin (1 µg/mL) were used as a positive control.

### 2.7 Calcium influx analysis

Calcium influx was assessed by flow cytometry using the ratiometric calcium indicator Indo-1. PBMCs isolated from healthy donors were seeded in a 6-well culture plate at a density of 5 × 10^6^ cells/mL and incubated with either an IgG control antibody or UMCD6 (10 μg/mL) for 24 hours. Following treatment, PBMCs were loaded with 2 μg/ml Indo-1 AM (Thermo Fisher Scientific, Cat# I1223) for 45 minutes, washed, and resuspended in HBSS supplemented with 1% FBS, 1 mM CaCl₂, 1 mM MgCl₂, and 10 mM HEPES. Cells were then stained with an FVD along with anti-CD4-PE/Cy7, anti-CD8-PerCP/Cy5.5, anti-CD56-APC/Cy7, anti-CD14-APC/Cy7, anti-CD16-APC/Cy7, and anti-CD19-APC/Cy7 (BioLegend). The fluorescence intensities of calcium-bound Indo-1 (excitation: 355 nm, emission: 372 nm) and calcium-free Indo-1 (excitation: 355 nm, emission: 514 nm) were measured using a Cytek Aurora. Cells were maintained at 4 °C and subsequently warmed to 37 °C in a bead bath for 15 minutes prior to flow cytometric analysis. Samples were first acquired for 30 seconds to establish baseline intracellular calcium levels. To assess calcium mobilization in response to TCR stimulation, cells were removed from the flow cytometer, stimulated by addition of anti-CD3 and anti-CD28 antibodies (5 μg/mL each) directly to a cell suspension, and then re-acquired on the flow cytometer for 10 minutes. To confirm the calcium mobilization capacity of the cells, ionomycin (1 μg/mL) was added to the cell suspension 10 minutes after the start of data acquisition, and data were collected for an additional 2 minutes. A calcium flux curve was generated using FlowJo software by calculating the time-dependent ratio of calcium-bound Indo-1 MFI to calcium-free Indo-1 MFI.

### 2.8 Western blotting

Cells were lysed using NE-PER™ Nuclear and Cytoplasmic Extraction Reagents (Thermo Fisher Scientific, Cat# 78833) supplemented with Protease Inhibitor Cocktail (Thermo Scientific, Cat# 78410) and Phosphatase Inhibitor Cocktail (Sigma-Aldrich, Cat# P0001-1ML). Protein concentrations were determined with the Pierce BCA Protein Assay Kit (Thermo Fisher Scientific, Cat# 23225) according to the manufacturer’s instructions. Equal amounts of nuclear protein (5-10 μg) were mixed with 4× Laemmli sample buffer (Bio-Rad, Cat# 1610747) containing β-mercaptoethanol, boiled for 5 min at 100 °C, and separated by SDS-PAGE on 4-20% Mini-PROTEAN^®^ TGX™ Precast Protein Gels (Bio-Rad, Cat# 4561094). Proteins were transferred onto nitrocellulose membranes using the iBlot™ 2 Dry Blotting System (Invitrogen). Membranes were blocked in 5% BSA in TBS (Bio-Rad, Cat# 1706435) with 0.1% Tween-20 for 1 hour at room temperature and then incubated overnight at 4 °C with primary antibodies against NFATC1 (1:1000, Invitrogen, Cat# MA3-024), GAPDH (1:10000, Cell Signaling Technology, Cat# 2118), or Histone H3 (1:1000, Cell Signaling Technology, Cat# 9715). After washing with TBST, membranes were incubated with HRP-conjugated secondary antibodies—anti-rabbit IgG-HRP (1:2000, Cell Signaling Technology, Cat# 7074) or anti-mouse IgG-HRP (1:2000, Cell Signaling Technology, Cat# 7076), for 1 hour at room temperature. Protein bands were visualized with enhanced chemiluminescence (SuperSignal™ West Dura Extended Duration Substrate, Thermo Fisher Scientific, Cat# PI34076) and imaged using the iBright CL1500 Imaging System (Thermo Fisher Scientific). Band intensities were quantified with ImageJ software (v1.54), and relative expression levels were normalized to Histone H3.

### 2.9 Phospho-flow cytometry

PBMCs pretreated with UMCD6 or an isotype-matched IgG control antibody (10 μg/mL) for 24 hours were stained with an FVD prior to activation. These cells were then stimulated with a combination of plate-bound anti-CD3 antibody and soluble anti-CD28 antibody (5 µg/mL) for the times indicated in the figure legends. Following stimulation, the cells were immediately fixed by adding BD Cytofix Buffer prewarmed at 37 °C directly to the cell culture and incubating for 15 minutes at 37 °C. After fixation, surface staining was performed using anti-CD3 Pacific Blue, anti-CD4 PE/Cy7, and anti-CD8α PerCP/Cy5.5. The cells were then permeabilized with BD™ Phosflow Perm Buffer III (BD Biosciences, Cat# 558050) prechilled at -20 °C. Finally, intracellular signaling molecules were stained with the antibodies listed in **Table 1**.

### 2.10 Statistical analysis

The statistical analysis was performed using GraphPad Prism (v10.4.1). Paired student’s t test was used for comparisons between two paired groups. Time-course data without missing values were analyzed using two-way repeated-measures ANOVA, with treatment and time as within-donor factors. For time-course datasets containing missing values, a mixed-effects model fitted by restricted maximum likelihood (REML), with treatment and time as fixed effects and donor as a random effect, was used instead. Both analyses were followed by Šídák’s multiple-comparisons test comparing IgG- and UMCD6-treated cells at each time point. The Geisser–Greenhouse correction was applied when the time factor included more than two levels. All statistical tests were two-sided, and P values < 0.05 were considered to be statistically significant.

## 3 Results

### 3.1 UMCD6 remodels the costimulatory receptor landscape in T cells

Because CD6 contributes to immune synapse organization through ligand-dependent adhesion and signaling, we first asked whether UMCD6 alters the expression of additional receptors that regulate T-cell activation and costimulation. Rather than broadly assuming suppression of TCR signaling, we reasoned that UMCD6 might modulate the molecular architecture of the immunological synapse and thereby alter the balance of activating signals.

**Figure 1:**
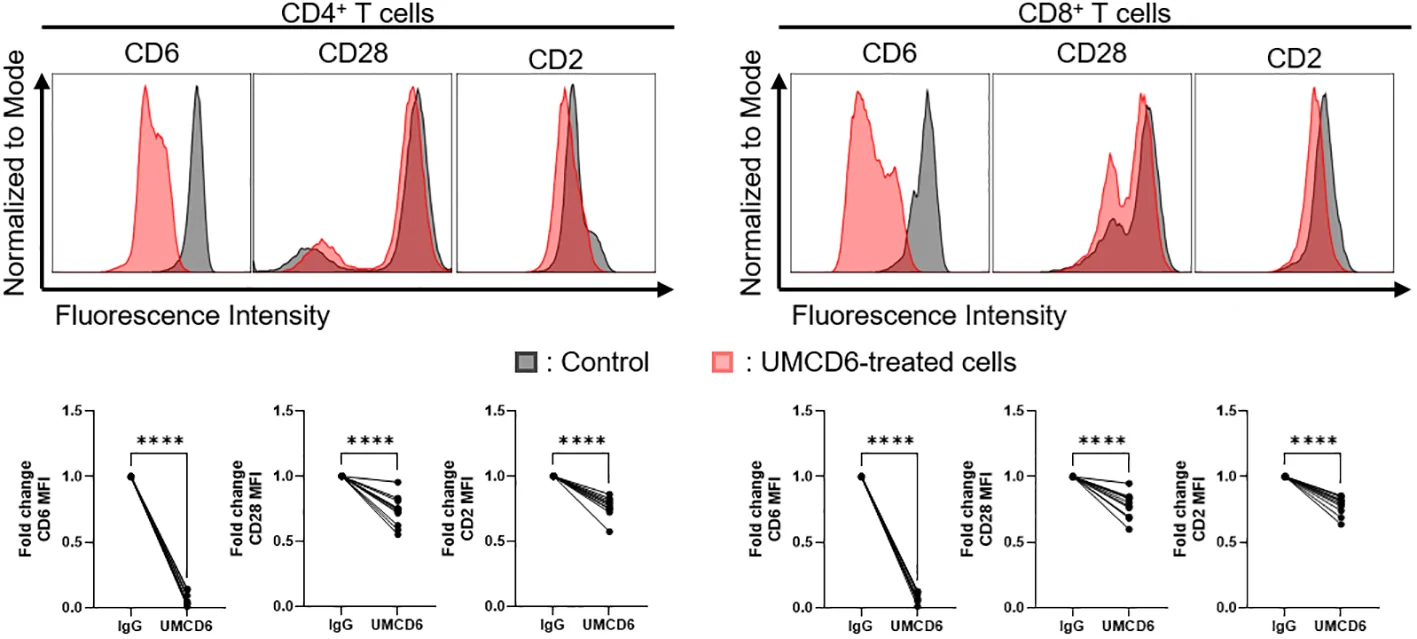
UMCD6 remodels the T-cell costimulatory receptor landscape and selectively reduces surface CD6, CD2, and CD28. Surface expression of CD6, CD2, and CD28 on CD4^+^ T cells (CD3^+^CD4^+^CD8^-^) and CD8^+^ T cells (CD3^+^CD4^-^CD8^+^) following 48 h treatment with IgG or UMCD6 was assessed by flow cytometry. MFI values were normalized within each donor to the paired IgG-treated control. Data presented as normalized MFI (fold change relative to IgG, where IgG = 1). Representative histograms and paired donor data from 10 independent donors are shown. Statistical significance was determined by paired t-test. ****P < 0.0001.

To address this, we first quantified mRNA expression of *CD6*, *CD28*, and *CD2* in peripheral blood mononuclear cells (PBMCs) following UMCD6 treatment. UMCD6 did not significantly alter *CD6* or *CD28* transcript levels, whereas *CD2* mRNA was significantly reduced (**Supplementary Figure 2**). We next assessed surface expression of these molecules on CD4^+^ and CD8^+^ T cells by flow cytometry. In both CD4^+^ and CD8^+^ T cells, UMCD6 significantly reduced surface expression of CD6, CD28, and CD2 (**Figure 1**). Together, these findings indicate that UMCD6 rapidly remodels the surface receptor profile of T cells, resulting in reduced expression of multiple costimulatory receptors.

### 3.2 UMCD6 promotes redistribution and internalization of CD6-associated costimulatory receptors

**Figure 2:**
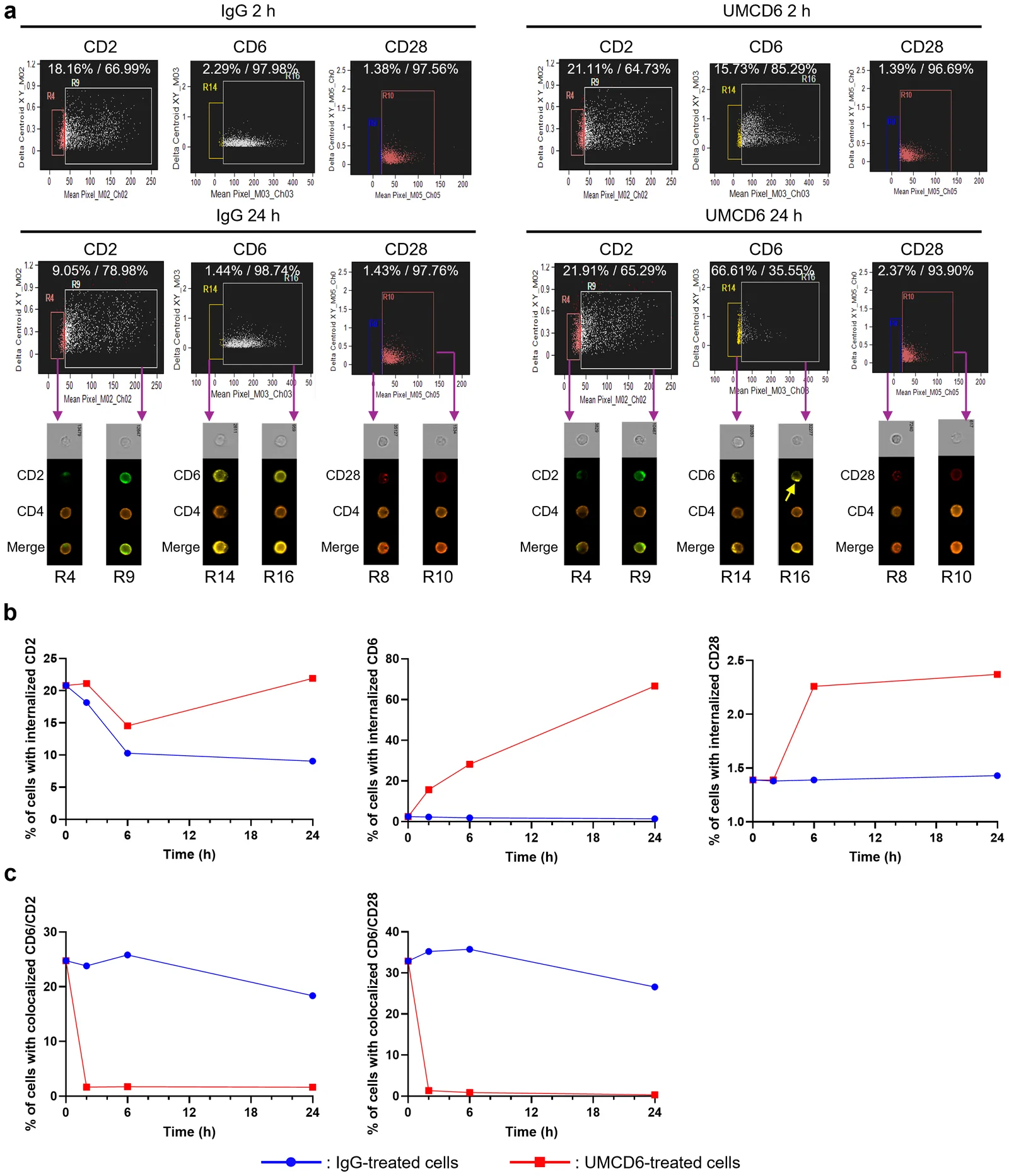
UMCD6 induces rapid internalization of CD6 and remodels the distribution of CD2 and CD28. **(a)** Representative imaging flow cytometry analysis of PBMCs treated with isotype-matched control IgG or UMCD6 for 2 h or 24 h. CD2^+^, CD6^+^, or CD28^+^ single-positive cells within the CD4^+^CD8^-^ population were gated and analyzed using Delta Centroid XY and Mean Pixel features. Cells with low Delta Centroid XY and low Mean Pixel values were defined as cells exhibiting greater internalization of each marker (gate R4 for CD2, gate R14 for CD6, and gate R8 for CD28), whereas cells with higher values were classified as less-internalized cells (gate R9 for CD2, gate R16 for CD6, and gate R10 for CD28). Representative gated populations and corresponding single-cell images from IgG- or UMCD6-treated cells at 24 h are shown. Yellow arrow indicates a site of polarized CD6 accumulation at the plasma membrane in UMCD6-treated cells with less CD6 internalization. **(b)** Time-course quantification of the percentage of cells exhibiting internalized CD2, CD6, or CD28 following treatment with IgG or UMCD6. **(c)** Time-course analysis of CD6-CD2 and CD6-CD28 colocalization following treatment with IgG or UMCD6. Blue and red lines denote IgG- and UMCD6-treated cells, respectively.

To determine whether the loss of surface CD6, CD2, and CD28 reflected receptor internalization and/or altered subcellular localization, we next analyzed their intracellular distribution by imaging flow cytometry. CD2^+^, CD6^+^, or CD28^+^ single-positive cells within the CD4^+^CD8^-^ population were gated and analyzed using Delta Centroid XY and Mean Pixel features. Cells with low Delta Centroid XY and low Mean Pixel values were defined as cells exhibiting greater internalization of each marker (gate R4 for CD2, gate R14 for CD6, and gate R8 for CD28), whereas cells with higher values were classified as less internalized cells (gate R9 for CD2, gate R16 for CD6, and gate R10 for CD28). Compared with IgG-treated controls, UMCD6-treated cells exhibited a higher frequency of cells classified as having internalized CD2 (21.91% vs 9.05%), CD6 (66.61% vs 1.44%), and CD28 (2.37% vs 1.43%) after 24 hours of treatment (**Figure 2a**). Representative images illustrating cells with and without CD6 internalization are shown in **Supplementary Figure 3**. Qualitative image analysis further revealed marked differences in CD6 membrane organization. In IgG-treated cells, even cells classified as “internalized” (gate R14 in the left panel of **Figure 2a**; **Supplementary Figure 3a**) generally retained a ring-like distribution of CD6 along the plasma membrane. In contrast, UMCD6-treated cells showed a clear disruption of this ring-like pattern (gate R14 in the right panel of **Figure 2a**; **Supplementary Figure 3b**). Among cells categorized as non-internalized (gate R16), CD6 remained uniformly distributed around the membrane in most IgG-treated cells, whereas in UMCD6-treated cells CD6 frequently lost its even membrane distribution and instead became concentrated at a single cellular pole (yellow arrow in **Figure 2a**), consistent with polarized redistribution preceding or accompanying internalization. Time-course analysis over 24 hours confirmed that the frequencies of CD2-, CD6-, and CD28-internalized cells remained consistently higher in UMCD6-treated samples than in IgG-treated controls (**Figure 2b**).

To determine whether these molecules remained associated during this process, we next assessed colocalization of CD2-CD6 and CD28-CD6. UMCD6 significantly reduced the proportion of cells showing CD2-CD6 and CD28-CD6 colocalization, indicating that UMCD6 not only alters receptor abundance at the cell surface but also disrupts the spatial organization of CD6-associated costimulatory complexes (**Figure 2c**). Collectively, these findings suggest that UMCD6 induces coordinated remodeling of the T-cell membrane, characterized by redistribution and internalization of key costimulatory receptors and loss of their normal association with CD6.

### 3.3 UMCD6 suppresses CD28-associated transcriptional output and reduces effector cytokine production

**Figure 3:**
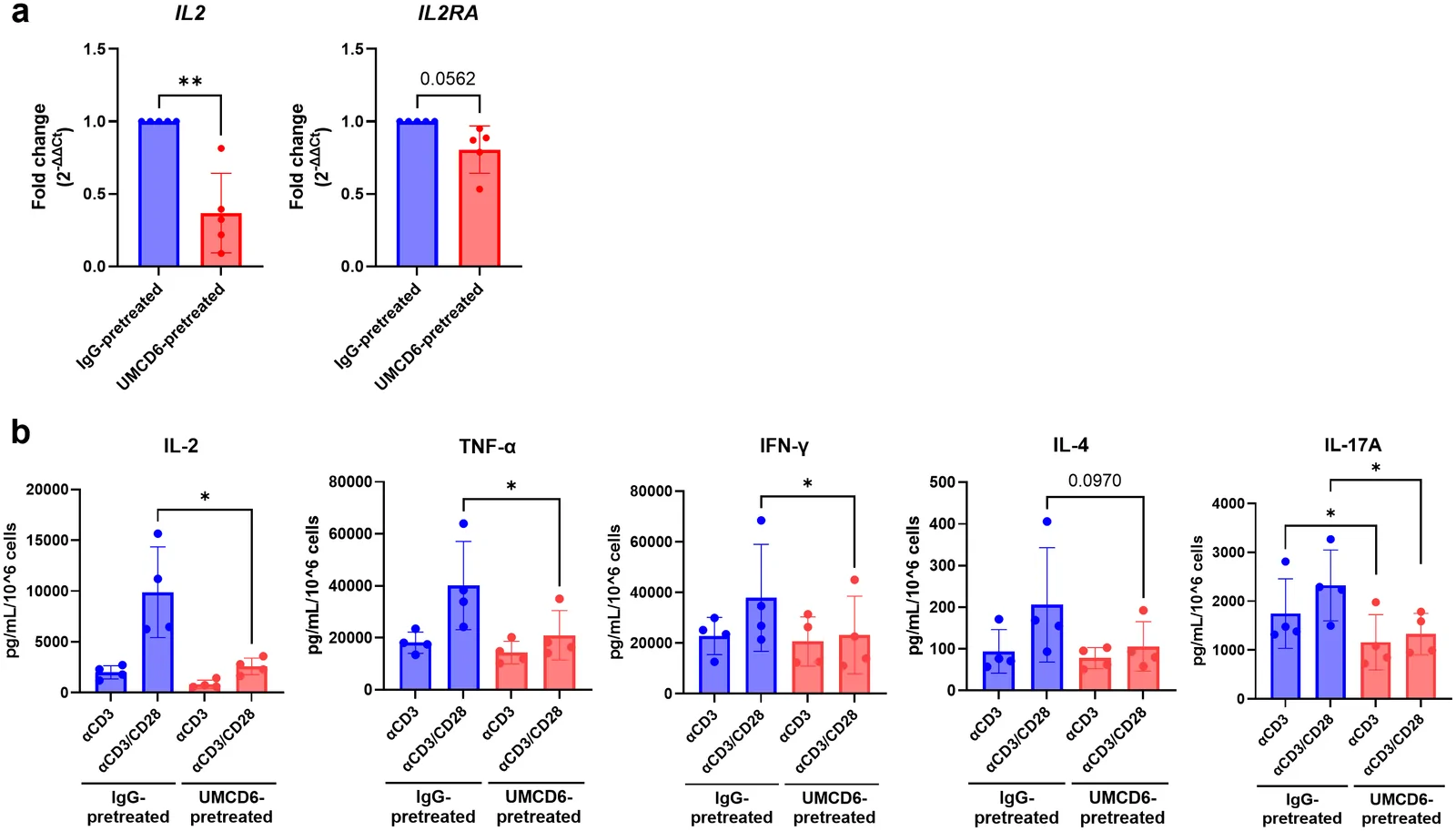
UMCD6 suppresses IL-2 pathway induction and inflammatory cytokine production following T-cell activation. **(a)** Relative mRNA expression of *IL2* and *IL2RA* in activated PBMCs pretreated with isotype-matched control IgG or UMCD6. PBMCs from healthy donors were pretreated for 24 h, then stimulated with plate-bound anti-CD3 plus soluble anti-CD28 for an additional 24 h. Gene expression was measured by qRT-PCR, normalized to β-actin, and calculated using the 2^-ΔΔCt^ method. Data are presented as fold change relative to the IgG-pretreated group (n = 5 donors). **(b)** Cytokine concentrations in culture supernatants from IgG- or UMCD6-pretreated PBMCs following activation with anti-CD3 alone or anti-CD3 plus anti-CD28. PBMCs were pretreated for 24 h, then stimulated for 24 h (IL-2) or 48 h (TNF-α, IFN-γ, IL-4, and IL-17A). Supernatants were analyzed by ELISA, and cytokine concentrations were normalized to cell number at the time of collection (n = 4 donors). Error bar shows standard deviation. Statistical significance was determined by paired t-test. *P < 0.05, **P < 0.01.

Because CD28 signaling amplifies TCR-driven activation by promoting the transcriptional activity of NFAT, NF-κB, and AP-1 (26), we next asked whether UMCD6 functionally attenuates CD28-dependent transcriptional programs. Following stimulation with plate-bound anti-CD3 and soluble anti-CD28 antibodies, PBMCs pretreated with UMCD6 exhibited significantly lower *IL2* mRNA expression than IgG-pretreated controls. *IL2RA* mRNA also trended lower, although this did not reach statistical significance (p = 0.0562) (**Figure 3a**). These data suggest that UMCD6 dampens early transcriptional responses associated with T-cell activation and IL-2 responsiveness.

We next assessed whether these transcriptional changes translated into altered cytokine production. PBMCs from healthy donors were pretreated with UMCD6 or control IgG, stimulated with anti-CD3/CD28 antibodies, and culture supernatants were analyzed by ELISA. UMCD6 pretreatment significantly reduced secretion of IL-2, TNF-α, IFN-γ, and IL-17A, whereas IL-4 production was not significantly affected (**Figure 3b**). This cytokine profile is consistent with selective suppression of proinflammatory and type 1/type 17 effector responses rather than global cytokine blockade, and it aligns with our prior observations that CD6 targeting restrains pathogenic Th1/Th17 programs.

### 3.4 UMCD6 attenuates CD4^+^ T-cell activation more strongly than CD8^+^ T-cell activation

**Figure 4:**
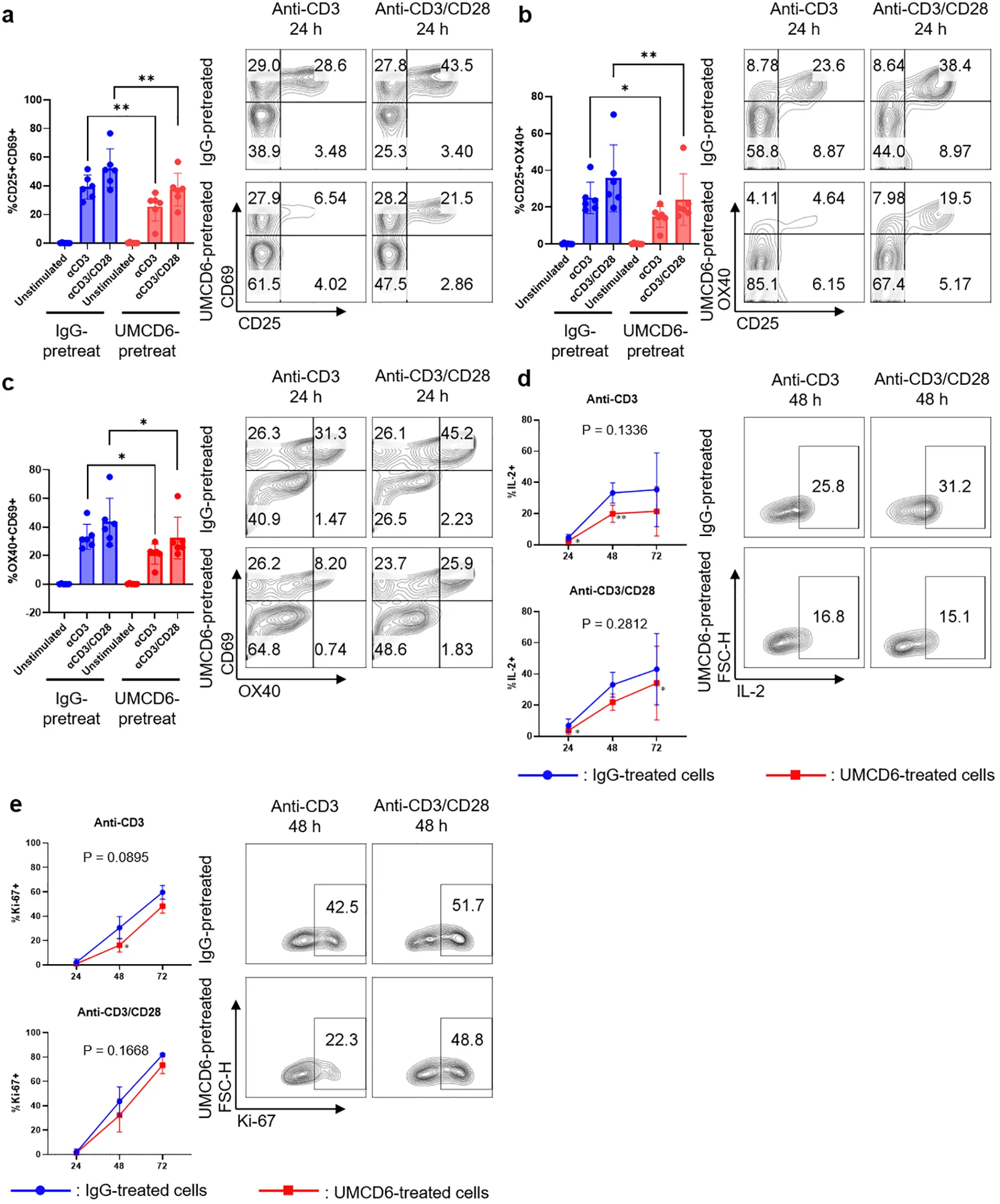
UMCD6 preferentially attenuates activation and cytokine induction in CD4+ T cells. **(a–c)** Frequencies of **(a)** CD25^+^CD69^+^, **(b)** CD25^+^OX40^+^, and **(c)** CD69^+^OX40^+^ cells among CD4^+^ T cells after 24 h of stimulation. PBMCs from healthy donors were pretreated with isotype-matched control IgG or UMCD6 for 24 h, then stimulated with plate-bound anti-CD3 alone or anti-CD3 plus soluble anti-CD28 for an additional 24 h. Representative flow cytometry plots and paired donor data are shown (n = 6). **(d)** Time-course analysis of intracellular IL-2^+^ CD4^+^ T cells following stimulation with anti-CD3 with or without anti-CD28 after isotype-matched control IgG or UMCD6 pretreatment. Representative flow cytometry plots at 48 h are shown. **(e)** Time-course analysis of Ki-67^+^ CD4^+^ T cells following stimulation with anti-CD3 with or without anti-CD28 after IgG or UMCD6 pretreatment. Representative flow cytometry plots at 48 h are shown. Blue and red lines indicate IgG- and UMCD6-pretreated cells, respectively. For the time-course analyses, n = 6 at 24 h and n = 4 at 48 and 72 h. Error bars shows standard deviation. For panels **(a–c)**, statistical significance was assessed using a paired t-test comparing control IgG- and UMCD6-pretreated cells within the corresponding stimulation condition. For panels **(d)** and **(e)**, each stimulation condition was analyzed separately using a mixed-effects model fitted by restricted maximum likelihood (REML), with treatment and time as fixed effects and donor as a random effect, followed by Šídák’s multiple-comparisons test comparing control IgG- and UMCD6-pretreated cells at each time point. The treatment × time interaction P value is shown for each time-course analysis. In panels **(a–c)**, asterisks indicate paired t-test comparisons; in panels **(d)** and **(e)**, asterisks indicate Šídák-adjusted comparisons between control IgG- and UMCD6-pretreated cells at the same time point. *P < 0.05, **P < 0.01.

To determine whether UMCD6 functionally suppresses T-cell activation downstream of anti-CD3/CD28 stimulation, we next examined canonical activation markers in CD4^+^ T cells, including CD25, CD69, and OX40. Time-course analyses of single-positive populations are shown in **Supplementary Figure 4**. Following anti-CD3/CD28 stimulation, UMCD6-pretreated CD4^+^ T cells displayed significantly reduced frequencies of CD25^+^CD69^+^, CD25^+^OX40^+^, and CD69^+^OX40^+^ double-positive cells compared with IgG-pretreated controls (**Figures 4a–c**). We next examine time-course changes of intracellular expression of IL-2 and Ki-67 following activation. In both CD4^+^ T cells stimulated with CD3 alone and those stimulated with anti-CD3/CD28 antibody, intracellular IL-2 expression levels were significantly lower at several time points in the UMCD6 pre-treated cells (**Figure 4d**). For Ki-67, a lower frequency of Ki-67^+^ CD4^+^ T cells was observed at 48 h following anti-CD3 stimulation, whereas no significant differences were detected at individual time points following anti-CD3/CD28 stimulation (**Figure 4e**). The treatment × time interactions for IL-2 and Ki-67 were not statistically significant under either stimulation condition. UMCD6 also reduced selected activation-associated readouts following anti-CD3 stimulation without exogenously added anti-CD28. However, because these experiments were performed in PBMC cultures, this condition did not exclude endogenous costimulatory signals provided by antigen-presenting cells and other accessory cells. Therefore, the observed suppression cannot be attributed specifically to direct modulation of TCR-CD6 signaling in the absence of costimulation. In contrast, the suppressive effects of UMCD6 on CD8^+^ T-cell activation were comparatively limited. Although CD25 expression was significantly reduced, the other activation markers were largely unchanged (**Supplementary Figures 5a, b**). To directly compare the effects of UMCD6 between CD4^+^ and CD8^+^ T cells, we performed subset × treatment interaction analyses using paired data from the same donors. Significant subset × treatment interactions were observed for the percentages of CD25^+^, CD69^+^, OX40^+^, and IL-2^+^ cells, with larger inhibitory effects in CD4^+^ than in CD8^+^ T cells (**Supplementary Figure 5c**). In contrast, the subset × treatment interaction was not significant for Ki-67^+^ cells. These findings provide direct statistical evidence that the effects of UMCD6 on activation-marker expression and IL-2 production are more pronounced in CD4^+^ than in CD8^+^ T cells.

### 3.5 UMCD6 preserves proximal TCR-driven calcium signaling

**Figure 5:**
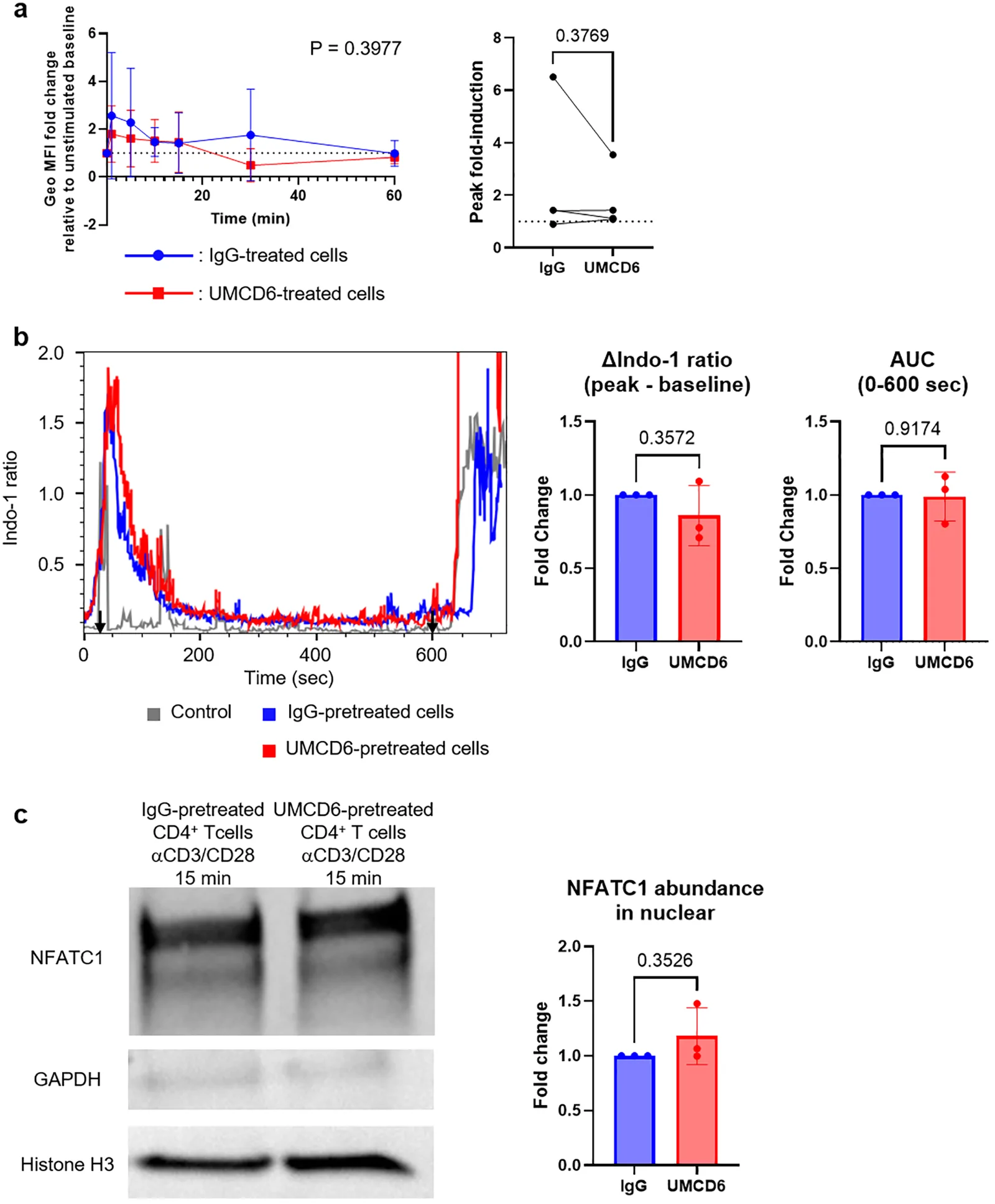
Limited effects of UMCD6 on LAT phosphorylation, calcium mobilization, and nuclear NFATC1 abundance in activated CD4+ T cells. **(a)** Time-course analysis of LAT phosphorylation in CD4^+^ T cells following anti-CD3/CD28 stimulation. PBMCs from healthy donors were pretreated with isotype-matched control IgG or UMCD6 for 24 h, then stimulated with plate-bound anti-CD3 plus soluble anti-CD28 for the indicated times. Cells were immediately fixed, surface stained, methanol permeabilized, and analyzed by phospho-flow cytometry. LAT phosphorylation was assessed by phospho-flow cytometry and expressed as the fold change in Geo MFI relative to the corresponding unstimulated baseline for each donor and treatment condition. Peak fold induction was calculated for each donor as the maximum Geo MFI among the post-stimulation time points divided by the corresponding baseline Geo MFI. Time-course data are presented as mean ± SD from four biologically independent donors. Peak fold-induction values from the same donor are connected by lines, and the horizontal dotted line indicates a fold induction of 1. Time-course data were analyzed using two-way repeated-measures ANOVA, with treatment and time as within-donor factors, followed by Šídák’s multiple-comparisons test comparing control IgG- and UMCD6-pretreated cells at each time point. The treatment × time interaction P value is shown. Peak fold-induction values were compared using a paired t test (P = 0.3769). **(b)** Intracellular Ca^2+^ flux in CD4^+^ T cells following isotype-matched control IgG or UMCD6 pretreatment. A representative kinetic curve of the Indo-1 ratio (Ca^2+^-bound/Ca^2+^-free) is shown. PBMCs were loaded with Indo-1, baseline fluorescence was recorded for 30 s, and anti-CD3 plus anti-CD28 was added (left arrow), followed by ionomycin (right arrow). The ΔIndo-1 ratio (peak - baseline) and area under the curve (AUC; 0–600 s) were quantified (n = 3 donors). **(c)** NFATC1 abundance in activated CD4^+^ T cells. Purified CD4^+^ T cells from healthy donors were pretreated with isotype-matched control IgG or UMCD6 for 24 h, stimulated with anti-CD3/anti-CD28 for 15 min, and nuclear extracts were analyzed by immunoblotting. NFATC1 signal was normalized to Histone H3 (n = 3 donors). Error bar shows standard deviation. No significant differences were detected between IgG- and UMCD6-treated groups in any panel (paired t-test).

To determine whether UMCD6 broadly impairs proximal TCR signaling, we next assessed LAT phosphorylation as a proximal TCR-associated signaling readout. Geo MFI of phospho-LAT was normalized to the corresponding unstimulated baseline for each donor and treatment condition. Anti-CD3/CD28 stimulation induced a modest and donor-variable increase in LAT phosphorylation in both control IgG- and UMCD6-pretreated CD4^+^ T cells (**Figure 5a**, left panel). Two-way repeated-measures ANOVA revealed no significant treatment × time interaction, and no significant differences between the treatment groups were detected at individual time points after adjustment for multiple comparisons (p = 0.3977). Peak fold-induction, calculated as the maximum post-stimulation Geo MFI relative to the corresponding baseline value, also did not differ significantly between control IgG- and UMCD6-pretreated cells (p = 0.3769) (**Figure 5a**, right panel). Thus, under the conditions examined, UMCD6 pretreatment did not detectably alter the stimulus-induced LAT phosphorylation response. We next evaluated intracellular calcium flux in activated CD4^+^ T cells using the ratiometric calcium indicator Indo-1. In three paired donors, no statistically significant differences were detected between UMCD6- and IgG-pretreated cells in calcium-flux kinetics, the change in the Indo-1 ratio from baseline to peak, or AUC values (**Figure 5b**). Finally, we assessed NFATC1 abundance in nuclear extracts as an orthogonal readout of Ca^2+^-calcineurin-NFAT pathway engagement. No significant difference was observed between the treatment groups, indicating that UMCD6 pretreatment did not detectably alter the accumulation of total NFATC1 in the nuclear-enriched fraction following CD4^+^ T-cell activation (**Figure 5c**; uncropped blot images were shown in **Supplementary Figure 6**). Together, these data did not reveal a large or consistent effect of UMCD6 on the calcium-signaling and nuclear NFATC1 readouts examined under the present experimental conditions.

### 3.6 UMCD6 modestly reduces the PKC-θ phosphorylation response following T-cell stimulation

Because the preceding analyses did not reveal a large or consistent effect of UMCD6 on the LAT phosphorylation, calcium-flux, or nuclear NFATC1 readouts examined, we next tested whether UMCD6 preferentially signaling molecules involved in costimulatory amplification. We therefore analyzed phosphorylation of key signaling intermediates associated with costimulatory amplification, including PKC-θ, which is recruited to the immunological synapse in a CD28-dependent manner (28–30), PI3K and Akt, and downstream transcriptional regulators within the integrated TCR/CD28 signaling network, including NF-κB, c-Fos, and c-Jun (27).

**Figure 6:**
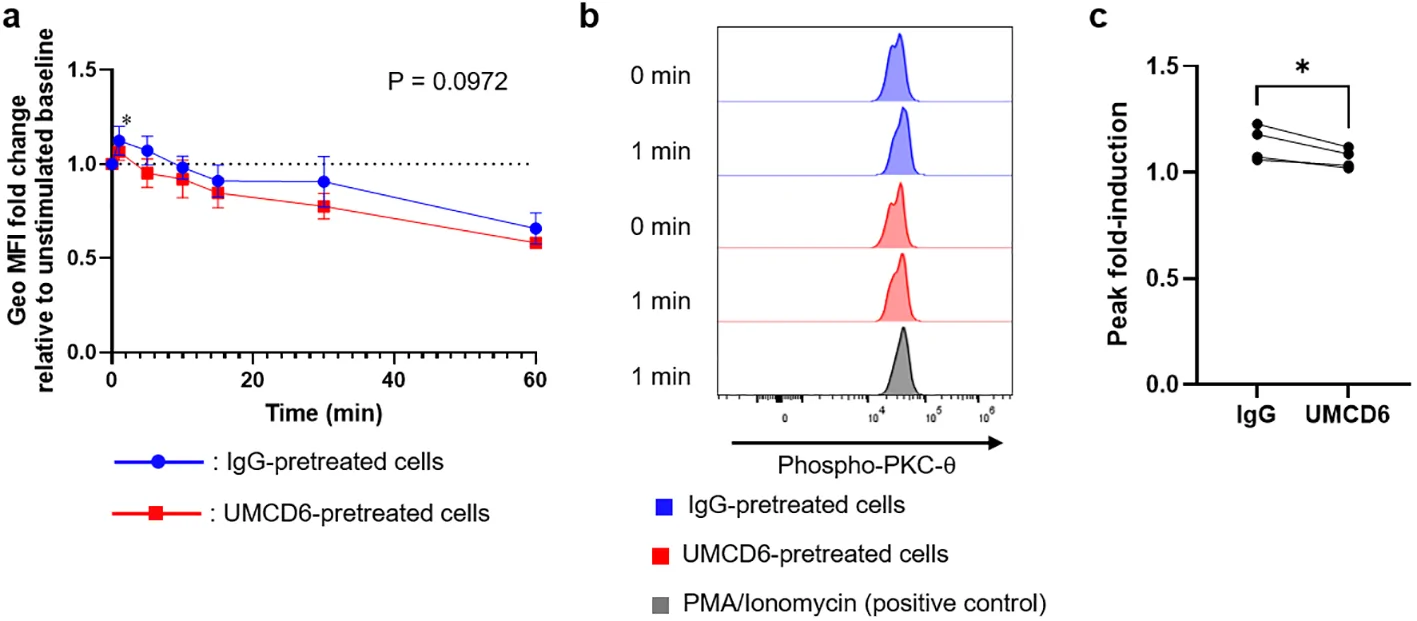
UMCD6 modestly reduces PKC-θ phosphorylation response in activated CD4+ T cells. **(a)** Time-course phospho-flow cytometric analysis of PKC-θ in CD4^+^ T cells following UMCD6 treatment. PBMCs from healthy donors were pretreated with isotype-matched control IgG or UMCD6 for 24 h, then stimulated with plate-bound anti-CD3 plus soluble anti-CD28 for the indicated times. Cells were immediately fixed to preserve phosphorylation, surface stained, methanol permeabilized, and stained with phospho-specific antibodies. For each donor and treatment condition, phospho-protein Geo MFI at each time point was normalized to the corresponding unstimulated value at 0 min. A fold change of 1 therefore represents the unstimulated baseline (n = 4 donors). Data were analyzed using two-way repeated-measures ANOVA followed by Šídák’s multiple-comparisons test. The treatment × time interaction P value is shown. Asterisks indicate Šídák-adjusted comparisons between IgG- and UMCD6-treated cells at the same time point. *P < 0.05. **(b)** Representative histograms showing PKC-θ phosphorylation before stimulation and at 1 minute after stimulation, corresponding to the early peak-response period. Blue and red denote IgG- and UMCD6-pretreated cells, respectively. Grey indicates PMA/Ionomycin treated cells. **(c)** Peak fold induction of PKC-θ phosphorylation. For each donor and treatment condition, fold induction was calculated by dividing the Geo MFI at each post-stimulation time point by the corresponding unstimulated Geo MFI, and the maximum value across the time course was defined as the peak fold induction. Paired values from the same donor are connected by lines and were compared using a paired t test. *P < 0.05.

Phospho-flow cytometric analysis showed a rapid increase in PKC-θ phosphorylation after anti-CD3/CD28 stimulation, with an early peak response at 1 minute (**Figures 6a, b**). Two-way repeated-measures ANOVA did not reveal a significant treatment × time interaction for baseline-normalized phospho-PKC-θ Geo MFI (p = 0.0972). Nevertheless, Šídák-adjusted multiple comparisons showed that the fold change relative to the corresponding unstimulated baseline was significantly lower in UMCD6-pretreated cells than in control IgG-pretreated cells at 1 min after stimulation. Moreover, the donor-level peak fold induction relative to the corresponding unstimulated baseline was significantly lower in UMCD6-pretreated cells than in IgG-pretreated cells (**Figure 6c**). These findings demonstrate a modest reduction in the early peak PKC-θ phosphorylation response following UMCD6 pretreatment.

The complete kinetic analyses of PKC-θ, PI3K, Akt, NF-κB, c-Fos, and c-Jun phosphorylation are presented in **Supplementary Figure 7**. For PI3K, Akt, NF-κB, and c-Fos, analyses of Geo MFI, baseline-normalized Geo MFI fold change, and the percentage of phospho-positive cells revealed neither significant treatment × time interactions nor significant Šídák-adjusted differences between the treatment groups at any individual time point. Their peak fold-induction values also did not differ significantly between control IgG- and UMCD6-pretreated cells (**Supplementary Figure 8**). For c-Jun, the percentage of phospho-c-Jun-positive cells showed a significant treatment × time interaction, whereas no significant difference in peak fold induction was observed. This finding indicates a treatment-associated change in the temporal pattern of c-Jun phosphorylation rather than a consistent reduction in its maximal response. Collectively, the reduction in peak PKC-θ fold induction represented the most consistent phospho-flow finding associated with UMCD6 pretreatment. Because PKC-θ integrates signals from the TCR and multiple costimulatory receptors, these findings are consistent with modulation of costimulatory signal amplification.

## 4 Discussion

**Figure 7:**
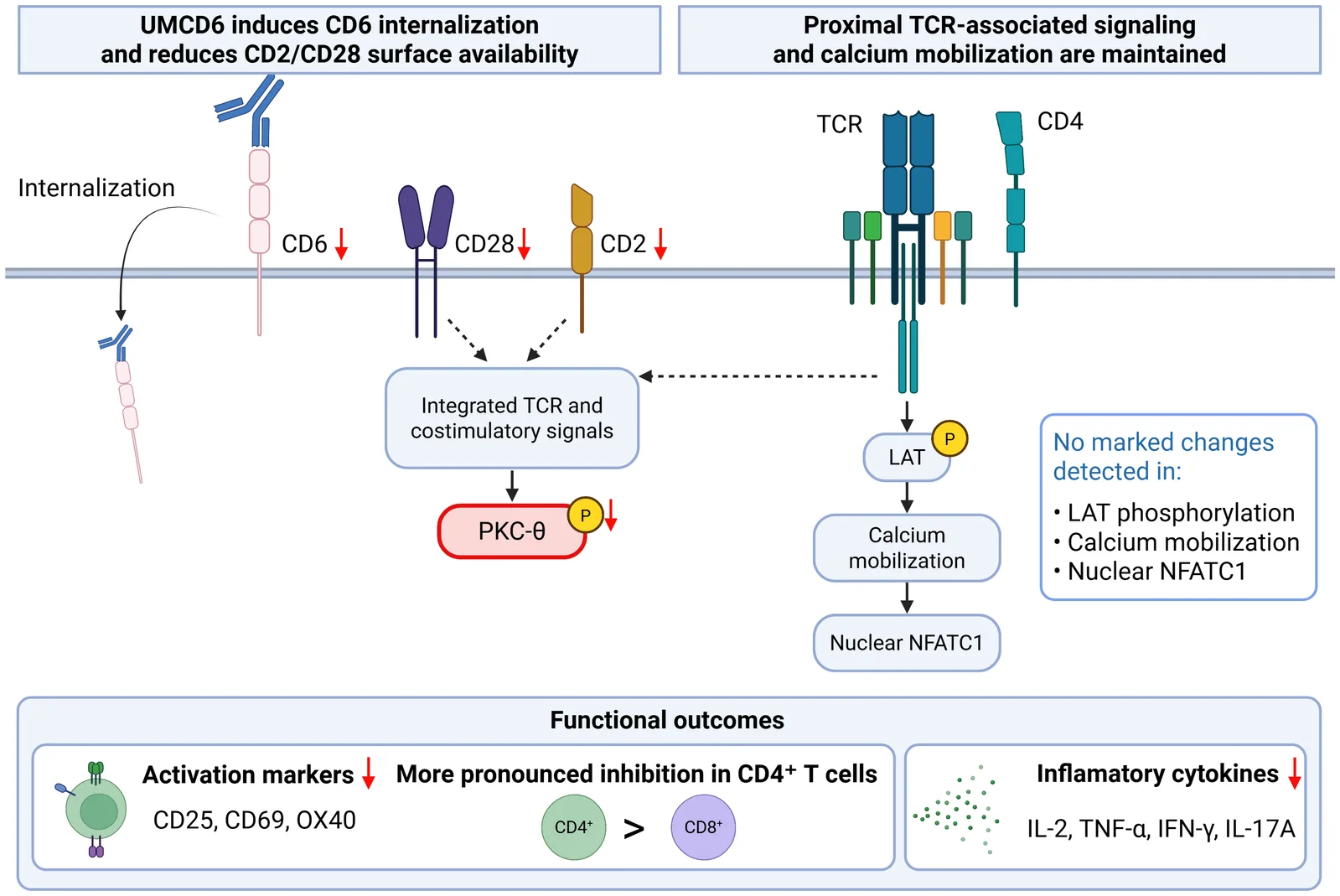
Proposed model of UMCD6-mediated suppression of T-cell activation. Schematic summary of the effects of UMCD6 on T-cell surface receptor organization, signaling, and functional responses. UMCD6 induces CD6 internalization and reduces the surface availability of the costimulatory molecules CD2 and CD28. Despite these changes, proximal T-cell receptor (TCR)-associated signaling, including LAT phosphorylation, calcium mobilization, and nuclear NFATC1, is largely maintained. In contrast, UMCD6 is associated with a modest reduction in the early peak fold induction of PKC-θ phosphorylation, consistent with altered costimulatory signal amplification. Functionally, UMCD6 suppresses the expression of activation markers, including CD25, CD69, and OX40, and reduces the production of inflammatory cytokines, including IL-2, TNF-α, IFN-γ, and IL-17A. These inhibitory effects are more pronounced in CD4+ T cells than in CD8+ T cells. Collectively, these findings support a model in which UMCD6 remodels the availability and organization of costimulatory receptors and preferentially suppresses CD4+ T-cell activation without broadly disrupting proximal TCR-associated signaling.

In the present study, we define a mechanistic framework by which the anti-CD6 monoclonal antibody UMCD6 restrains human T-cell activation. Using complementary phenotypic, imaging, transcriptional, and signaling analyses, we show that UMCD6 induces rapid remodeling of the T-cell surface receptor landscape, characterized by reduced surface expression and internalization of CD6 together with decreased availability of the costimulatory receptors CD2 and CD28. Functionally, these changes were associated with diminished induction of activation markers and suppression of pro-inflammatory cytokine production, particularly in CD4^+^ T cells. Among the phospho-flow readouts examined, a reduction in peak PKC-θ phosphorylation represented the most consistent signaling change associated with UMCD6 pretreatment. By contrast, the LAT phosphorylation, calcium-flux, and nuclear NFATC1 analyses did not reveal large or consistent treatment-associated differences. Collectively, these findings support a model in which UMCD6 does not extinguish TCR triggering per se, but instead selectively lowers the amplitude and durability of downstream costimulatory signaling required for full effector differentiation. These results are summarized in **Figure 7**.

A central observation in this study is that UMCD6 induces marked redistribution of CD6 on the plasma membrane, including receptor capping and internalization. This finding provides a mechanistic explanation for the reduction in surface CD6 detected by flow cytometry and argues against a purely technical explanation such as epitope masking alone. Although we cannot fully exclude contributions from receptor shedding or altered recycling, the imaging data strongly support active receptor reorganization as a major consequence of UMCD6 engagement. Given that the cytoplasmic tail of CD6 functions as a scaffold for signaling complexes assembled during T-cell activation, such spatial redistribution is likely to have consequences beyond simple loss of surface abundance. In particular, displacement of CD6 from its normal synaptic architecture may impair its ability to coordinate receptor proximity, sustain signaling microclusters, and stabilize productive T cell-APC interactions.

The effects of UMCD6 extended beyond CD6 itself. Surface expression of CD2 and CD28 was also reduced, and imaging flow cytometry demonstrated increased internalization of both receptors after UMCD6 treatment. Importantly, the limited colocalization observed between CD6 and either CD2 or CD28 argues against a simple model of passive co-internalization within a stable receptor complex. Instead, our data suggest that CD6 ligation by UMCD6 triggers broader remodeling of the costimulatory receptor network. This interpretation is further supported by the reduction in CD2 transcripts, indicating that at least part of the effect may involve secondary transcriptional or post-transcriptional regulation rather than receptor trafficking alone. Thus, UMCD6 appears to induce a coordinated decrease in costimulatory receptor availability that would be expected to reduce the efficiency of immune synapse maturation and limit the capacity of activated T cells to achieve full effector competence. However, a decrease in cell-surface molecule expression can result from various mechanisms other than internalization, including epitope masking, receptor shedding, altered recycling, and transcription-independent turnover. Further research is required to determine whether these mechanisms contribute to the inhibitory effects exerted by UMCD6.

The reduction in PKC-θ phosphorylation provides a potential mechanistic link between the altered surface phenotype induced by UMCD6 and the subsequent suppression of CD4^+^ T-cell activation. Following T-cell activation, PKC-θ is recruited to the immunological synapse formed between T cells and antigen-presenting cells, where it mediates the integration and propagation of activation signals (28–30). In the present study, UMCD6 reduced the surface expression of both CD2 and CD28, indicating decreased availability of molecules that contribute to the organization and function of the immunological synapse. Reduced availability of these synaptic costimulatory molecules may diminish the signaling competence of the T-cell–APC interface and thereby contribute to the lower peak PKC-θ phosphorylation observed following stimulation. However, we did not directly examine immunological-synapse formation, receptor microcluster organization, or the recruitment of PKC-θ to the T-cell–APC interface. Therefore, the proposed relationship between reduced CD2 and CD28 surface availability and modestly reduced PKC-θ signaling remains mechanistically plausible but unproven. Direct imaging of the immunological synapse will be required to test this proposed relationship.

The transcriptional and functional findings were also consistent with attenuation of productive costimulatory activation. UMCD6 reduced induction of *IL2* and trended toward suppression of *IL2RA* following anti-CD3/CD28 stimulation, and these changes were mirrored by reduced intracellular IL-2 and lower frequencies of activated CD25^+^CD69^+^, CD25^+^OX40^+^, and CD69^+^OX40^+^ CD4^+^ T cells. Because IL-2 production and IL-2 receptor upregulation are canonical readouts of productive costimulation (31–34), these findings provide a coherent mechanistic bridge between receptor remodeling, attenuation of downstream signaling, and impaired T-cell activation. The selective reduction in IL-2 likely has broader consequences, as IL-2 availability influences not only proliferation but also differentiation, survival, and the competitive fitness of activated T cells within inflammatory environments.

Our findings also refine the interpretation of CD6 as a signaling receptor. Beyond its adhesive role, CD6 is increasingly recognized as a cytoplasmic scaffold that recruits signaling mediators, including SLP-76, VAV1, and ZAP70-associated complexes, following TCR engagement (5). This scaffolding function has been proposed to operate in parallel with, rather than strictly downstream of, canonical LAT-dependent signaling. In the present experiments, UMCD6 pretreatment did not detectably reduce the stimulus-induced LAT phosphorylation response, calcium mobilization, or total NFATC1 abundance in nuclear-enriched fractions. Taken together, these concordant observations argue against broad inhibition of proximal TCR-Ca^2+^ signaling as the primary basis for the suppressive effects of UMCD6. Instead, when considered alongside the reduced surface availability of CD2 and CD28 and the attenuation of peak PKC-θ phosphorylation, the findings support a model in which UMCD6 predominantly alters CD6-associated synaptic organization and costimulatory signal amplification while leaving initial TCR-triggered responses largely intact. Although the modest LAT response and limited donor numbers preclude exclusion of smaller effects on proximal signaling, the overall signaling profile is more consistent with attenuation of costimulatory amplification than with global blockade of TCR signaling.

An important feature of the current study is that it also begins to explain the subset specificity of the functional effects of UMCD6. Direct subset × treatment interaction analyses demonstrated that the effects of UMCD6 on CD25, CD69, and OX40 expression and intracellular IL-2 production differed significantly between CD4^+^ and CD8^+^ T cells, with greater inhibition observed in CD4^+^ T cells. Differences in the organization of and dependence on costimulatory pathways may contribute to the greater sensitivity of conventional CD4^+^ T-cell activation. In healthy adults, CD28 expression is nearly universal among CD4^+^ T cells, whereas a substantial fraction of CD8^+^ T cells (particularly more differentiated or effector-memory populations) can be CD28 low or negative (35). Differences in CD6 expression, differentiation state, and dependence on CD2-, CD28-, and CD6-associated signaling may also influence the response to UMCD6. This interpretation is also consistent with our prior work showing that UMCD6 can enhance activation-associated and cytotoxic features in CD8^+^ T cells, including increased NKG2D expression and effector function. Taken together, these observations suggest that UMCD6 is not globally immunosuppressive, but instead exerts context- and subset-dependent immunomodulatory effects: it preferentially restrains costimulation-dependent helper T-cell activation while preserving, and in some settings enhancing, cytotoxic lymphocyte function.

Although these findings indicate preferential suppression of conventional CD4^+^ T-cell activation, they also raise an important unresolved question regarding regulatory T cells. In particular, the reduction in IL-2 production may have consequences for Treg biology, because IL-2 is required for Treg survival, STAT5 activation, FOXP3 stability, and suppressive function (36). Therefore, decreased IL-2 availability could theoretically impair Treg maintenance or function, even while limiting conventional effector CD4^+^ T-cell activation. Conversely, UMCD6 may also have direct effects on Tregs through CD6 engagement or through changes in CD2 and CD28 availability, and these effects may differ from those observed in conventional CD4^+^ T cells. The present study was not designed to define the impact of UMCD6 on Tregs, and we did not assess FOXP3^+^CD25^hiCD127^lo Treg frequency, Treg-specific signaling responses, suppressive capacity, or rescue with exogenous IL-2. Thus, the net effect of UMCD6 on the balance between effector and regulatory CD4^+^ T-cell responses remains to be determined in future studies.

This subset-selective activity has important translational implications. UMCD6 significantly reduced production of IFN-γ, IL-17A, TNF-α, and IL-2, cytokines central to pathogenic Th1- and Th17-skewed inflammation and strongly implicated in multiple autoimmune and chronic inflammatory diseases. By contrast, IL-4 was relatively preserved. Although the basis for this differential cytokine effect remains to be fully defined, the overall pattern is consistent with preferential attenuation of inflammatory helper T-cell programs rather than indiscriminate suppression of all T-cell function. In this context, our findings provide a mechanistic rationale for the therapeutic potential of CD6-directed therapies in diseases driven by aberrant Th1/Th17 responses.

The extracellular biology of CD6 may further influence the effects of UMCD6. CD6 interacts with ALCAM/CD166 and has also been reported to engage additional ligands, including CD318 and CD44 (3), depending on the cellular and experimental context. These ligand systems may differ in expression pattern, inflammatory regulation, and contribution to synapse formation or tissue localization. The present study was not designed to dissect ligand-specific mechanisms, and we cannot determine whether UMCD6 acts primarily by blocking ligand engagement, inducing receptor redistribution, promoting receptor internalization, or combining these effects. Similarly, we did not benchmark UMCD6 against other anti-CD6 antibodies such as itolizumab. Because anti-CD6 antibodies may differ in epitope specificity, ligand-blocking capacity, valency-dependent clustering, and downstream functional effects, no mechanistic or therapeutic comparisons between UMCD6 and other anti-CD6 antibodies can be drawn from the present data. Direct comparative studies will be needed before conclusions can be drawn regarding the relative mechanistic or therapeutic advantages of UMCD6.

The observation that UMCD6 partially suppressed activation even under anti-CD3 stimulation alone deserves particular consideration. Because these experiments were performed in PBMC cultures rather than purified T cells, activated T cells remained exposed to endogenous antigen-presenting cells and therefore to contact-dependent interactions mediated by CD6 ligands, CD80/CD86-CD28, and CD2-LFA-3 (37). Thus, this condition cannot be regarded as a costimulation-free system. The present experiments therefore cannot determine whether UMCD6 directly modulates T-cell-intrinsic TCR–CD6-associated signaling independently of CD28 or instead attenuates activation by altering endogenous accessory-cell interactions. Nevertheless, these findings demonstrate that UMCD6 can suppress selected activation responses in a multicellular PBMC environment in which endogenous costimulatory inputs remain available. Experiments using purified CD4^+^ and CD8^+^ T cells stimulated with plate-bound anti-CD3 will be required to distinguish these possibilities.

Several limitations should be acknowledged. First, PBMCs were pretreated with UMCD6 for 24 h before T-cell stimulation. Thus, the present findings primarily demonstrate the effects of UMCD6 on the subsequent activation of initially unstimulated T cells and do not establish whether UMCD6 can suppress T cells after activation has already begun. Time-of-addition experiments will be required to determine whether its inhibitory effects extend to ongoing T-cell responses. Second, although imaging flow cytometry demonstrated marked CD6 internalization, more modest CD2 and CD28 redistribution, the present study does not establish that receptor internalization is required for the functional inhibitory effects of UMCD6. The association among receptor redistribution, reduced surface receptor availability, altered signaling, and suppression of T-cell activation therefore remains correlative. Higher-resolution confocal/TIRF microscopy, direct T cell-APC synapse imaging, and experiments that selectively prevent receptor internalization will be needed to determine how these receptor-level changes affect microcluster organization, receptor residency, PKC-θ recruitment, and contact architecture at the T-cell–APC interface. Third, the present study does not resolve the relative contributions of ligand blockade, antibody-induced receptor clustering, epitope masking, receptor shedding, altered recycling, receptor turnover or degradation, or Fc receptor-dependent crosslinking. In particular, comparison of intact UMCD6 with Fab or F(ab’)2 fragments, Fc receptor blockade, and experiments using purified T cells or defined accessory-cell systems would be important to distinguish physiological CD6 modulation from antibody-induced clustering effects. Fourth, several mechanistic assays were performed using a limited number of biologically independent donors (n = 3-6), and no formal *a priori* power calculation was performed. In particular, the calcium-flux and nuclear NFATC1 analyses included three donors and were underpowered to exclude small or moderate effects of UMCD6. The stimulus-induced LAT phosphorylation response was also modest and variable among donors. Therefore, the absence of statistically significant differences in these assays should not be interpreted as evidence of equivalence or complete preservation of the signaling pathways examined. Fifth, the relative contributions of CD2 and CD28 to UMCD6-mediated modulation of costimulatory signaling remain unresolved. Because PKC-θ integrates signals from multiple receptors at the immunological synapse, the observed reduction in PKC-θ phosphorylation cannot be assigned to a single costimulatory pathway. Receptor-specific perturbation studies will be required to define the respective roles of CD2 and CD28. Sixth, the present experiments were performed at fixed anti-CD3 and anti-CD28 concentrations and therefore did not determine whether the magnitude of UMCD6-mediated suppression varies with TCR signal strength. Experiments using graded anti-CD3 stimulation or antigen-specific ligands of defined affinity will be required to establish whether UMCD6 preferentially inhibits responses that are more dependent on costimulatory amplification. Seventh, this study did not include broad transcriptomic profiling, comprehensive phenotyping, antigen-specific responses, APC-dependent activation assays, purified or polarized CD4^+^ T-cell subsets, Treg functional assays, or long-term proliferation and expansion measurements. These approaches will be important to define the physiological relevance and disease-specific consequences of UMCD6-mediated T-cell modulation. Finally, although our findings are consistent with prior *in vivo* observations, additional studies in disease-relevant human systems and orthogonal *in vivo* models will be necessary to establish how these signaling effects translate into therapeutic efficacy across distinct immune-mediated pathologies.

In summary, our findings support a model in which UMCD6 reprograms CD6-dependent immune synapse function by inducing receptor redistribution and coordinated loss of key costimulatory receptors, thereby altering PKC-θ-associated costimulatory signaling without broadly blocking the proximal T-cell receptor-associated responses. This mechanistic bias results in preferential inhibition of CD4^+^ T-cell activation and suppression of Th1/Th17-associated inflammatory outputs, while sparing, or in other settings potentially enhancing, cytotoxic lymphocyte functions. These data position UMCD6 as a mechanistically distinct CD6-directed immunomodulator and provide a framework for understanding how selective remodeling of synaptic costimulation may offer therapeutic benefit in autoimmunity, chronic inflammation, and other diseases in which pathogenic helper T-cell responses drive tissue injury. These findings raise the possibility that CD6 targeting may provide a therapeutic strategy that selectively restrains pathogenic helper T-cell responses while preserving important aspects of cytotoxic lymphocyte function.

## Data Availability

The original contributions presented in the study are included in the article/**Supplementary Material**. Further inquiries can be directed to the corresponding authors.
