# Supplementary material for: Anti-CD6 monoclonal antibody UMCD6 induces CD6 internalization, remodels costimulatory receptor availability, and modulates CD4+ T-cell activation
Source: Front Immunol. 2026 Aug 19;17:1918840. doi: 10.3389/fimmu.2026.1918840 (PMC13533903; doi:10.3389/fimmu.2026.1918840)
Supplement: Supplementary file 1 [file DataSheet1.docx]

**Supplementary Figure 1. Flow cytometry and imaging flow cytometry gating strategies.**

(a) Gating strategy used for analysis of surface receptor expression in Figure 1b.
(b) Gating strategy used for imaging flow cytometry analyses in Figure 2.
(c) Gating strategy used for the activation-induced marker (AIM) assay in Figure 4.
(d) Gating strategy used for the calcium flux assay in Figure 5b.
(e) Gating strategy used for phospho-flow cytometry analyses in Figures 5a and 6a-f.

**Supplementary Figure 2. UMCD6 selectively reduces *CD2* mRNA expression without affecting *CD6* or *CD28* mRNA expression.**

Relative mRNA expression of *CD6*, *CD2*, and *CD28* in PBMCs treated with isotype-matched control IgG or UMCD6 for 24 h. Total RNA was isolated and analyzed by qRT-PCR, and expression was normalized to β-actin using the 2^-ΔΔCt^ method. Data are presented as fold change relative to IgG-treated controls (n = 3 donors). Error bar shows standard deviation. Statistical significance was determined by paired t-test. *P < 0.05.

**Supplementary Figure 3. Representative imaging flow cytometry examples of CD6 surface retention versus internalization.**

Representative single-cell images illustrating internalization of CD6 after 24 h treatment with (a) IgG or (b) UMCD6, as defined by the imaging flow cytometry internalization mask used in Figure 2.

**Supplementary Figure 4. UMCD6 reduces activation responses in CD4⁺ T cells.**

Time-course analysis of the frequencies of CD25⁺, CD69⁺, and OX40⁺ cells among CD4⁺ T cells following stimulation with anti-CD3 alone, anti-CD3 plus anti-CD28, or no stimulation. PBMCs from healthy donors were pretreated with IgG or UMCD6 for 24 h prior to stimulation. Blue and red lines indicate IgG- and UMCD6-pretreated cells, respectively. n = 6 at 24 h and n = 4 at 48 and 72 h. Error bar shows standard deviation. Each stimulation condition was analyzed separately using a mixed-effects model fitted by restricted maximum likelihood (REML), with treatment and time as fixed effects and donor as a random effect, followed by Šídák’s multiple-comparisons test comparing control IgG- and UMCD6-pretreated cells at each time point. The treatment × time interaction P value is shown. Asterisks indicate Šídák-adjusted comparisons between control IgG- and UMCD6-pretreated cells at the same time point. *P < 0.05, **P < 0.01.

**Supplementary Figure 5. UMCD6 exerts comparatively limited effects on CD8⁺ T-cell activation.**

(a) Time-course analysis of the frequencies of CD25⁺, CD69⁺, OX40⁺, 4-1BB⁺, IL-2⁺, Ki-67⁺, and granzyme B⁺ cells among CD8⁺ T cells following stimulation with anti-CD3 alone, anti-CD3 plus anti-CD28, or no stimulation. PBMCs from healthy donors were pretreated with IgG or UMCD6 for 24 h before stimulation. Blue and red lines denote IgG- and UMCD6-pretreated cells, respectively (n = 4-6 donors). (b) Representative flow cytometry plots showing CD25 expression versus FSC-H in IgG- or UMCD6-pretreated CD8⁺ T cells after 24 h of stimulation. Error bar shows standard deviation. Statistical significance was determined by paired t-test comparing UMCD6 with IgG at the corresponding time point. *P < 0.05, **P < 0.01. (c) Frequencies of CD25⁺, CD69⁺, OX40⁺, intracellular IL-2⁺, and Ki-67⁺ cells among CD4⁺ and CD8⁺ T cells following anti-CD3 plus anti-CD28 stimulation. PBMCs from healthy donors were pretreated with UMCD6 or an isotype-matched control IgG for 24 h before stimulation. CD25, CD69, OX40, and intracellular IL-2 were assessed at 24 h after stimulation (n = 6), whereas Ki-67 was assessed at 48 h (n = 4). Individual donor values are shown, and paired control IgG- and UMCD6-treated samples within each T-cell subset are connected by lines. CD4⁺ and CD8⁺ T-cell measurements were obtained from the same donors. Differences in the effects of UMCD6 between T-cell subsets were assessed using two-way repeated-measures ANOVA, with subset and treatment as within-donor factors. The subset × treatment interaction P value is shown for each readout.

**Supplementary Figure 6. Original Western Blot membrane showing the effect of UMCD6 on NFATC1 phosphorylation in activated CD4^+^T cells.**

Purified CD4⁺ T cells from healthy donors were pretreated with IgG or UMCD6 for 24 h, stimulated with anti-CD3/anti-CD28 for 15 min, and nuclear extracts were analyzed by immunoblotting. The representative band in Figure 5c was cropped from the region indicated in red.

**Supplementary Figure 7. Kinetic analyses of PKC-θ, PI3K, Akt, NF-κB, c-Fos, and c-Jun phosphorylation in control IgG- and UMCD6-pretreated CD4^+^ T cells.**

(a-f) Time-course phospho-flow cytometric analysis of signaling intermediates in CD4^+^ T cells following UMCD6 treatment. PBMCs from healthy donors were pretreated with isotype-matched control IgG or UMCD6 for 24 h, then stimulated with plate-bound anti-CD3 plus soluble anti-CD28 for the indicated times. Cells were immediately fixed to preserve phosphorylation, surface stained, methanol permeabilized, and stained with phospho-specific antibodies. Spaghetti plots show Geo MFI, the percentage of phospho-positive cells within the CD3^+^CD4^+^CD8^-^ gate, and baseline-normalized Geo MFI fold change (n = 4 donors). Representative histograms are shown for each analyte, with numbers indicating stimulation time points. Blue and red denote IgG- and UMCD6-pretreated cells, respectively. Grey indicates PMA/Ionomycin treated cells. Data were analyzed using two-way repeated-measures ANOVA followed by Šídák’s multiple-comparisons test. The treatment × time interaction P value is shown. Asterisks indicate Šídák-adjusted comparisons between IgG- and UMCD6-treated cells at the same time point. *P < 0.05.

**Supplementary Figure 8. Peak fold induction of PI3K, Akt, NF-κB, c-Fos, and c-Jun phosphorylation in control IgG- and UMCD6-pretreated CD4^+^ T cells.**

PBMCs from healthy donors were pretreated with UMCD6 or an isotype-matched control IgG for 24 h and subsequently stimulated with anti-CD3 plus anti-CD28 antibodies. Phosphorylation of NF-κB, PI3K, Akt, c-Fos, and c-Jun was assessed in CD4^+^ T cells by phospho-flow cytometry. For each donor and treatment condition, peak fold induction was calculated by dividing the maximum post-stimulation Geo MFI observed across the analyzed time points by the corresponding unstimulated baseline Geo MFI. Individual values represent biologically independent donors (n = 4), and paired control IgG- and UMCD6-treated samples from the same donor are connected by lines. Differences between the treatment groups were assessed using paired t tests.
